# Comparison of viral and epidemiological profiles of hospitalized children with severe acute respiratory infection in Beijing and Shanghai, China

**DOI:** 10.1186/s12879-019-4385-5

**Published:** 2019-08-20

**Authors:** Yanjie Zhao, Roujian Lu, Jun Shen, Zhengde Xie, Gaoshan Liu, Wenjie Tan

**Affiliations:** 1 Key Laboratory of Laboratory Medicine, Ministry of Education, and Institute of Medical Virology, Wenzhou Medical University, Zhejiang, China; 2 National Institute for Viral Disease Control and Prevention, China CDC, 155Changbai Road, Beijing, 102206 Changping District China; 3 Children’s Hospital of Fudan University, Shanghai, China; 4 Key Laboratory of Major Diseases in Children and National Key Discipline of Pediatrics (Capital Medical University), Ministry of Education, Beijing Pediatric Research Institute, Beijing Children’s Hospital, Capital Medical University, Beijing, 100045 China

**Keywords:** Beijing, Children, Epidemiological profile, Nasopharyngeal aspirates, Polymerase chain reaction, Severe acute respiratory infection, Shanghai, Virus

## Abstract

**Background:**

No comparison data have been reported on viral and epidemiological profiles of hospitalized children with severe acute respiratory infection (SARI) in Beijing or Shanghai, China.

**Methods:**

We collected 700 nasopharyngeal aspirates (NPA) from hospitalized children with SARI in Beijing (northern China) and Shanghai (southern China). Multiple respiratory viruses (including 15 common viruses) were screened by validated polymerase chain reaction (PCR) or real-time reverse transcription-PCR assays and confirmed by sequencing. Demographic data and the distribution of viral infections were also examined.

**Results:**

Of 700 samples, 547 (78.1%) tested positive for viral infections. The picornaviruses (PIC), which included rhinovirus (RV) and enterovirus (EV), were the most common (34.0%), followed by respiratory syncytial virus (RSV) (28.3%), human bocavirus (HBoV) (19.1%), adenovirus (ADV) (13.7%), human coronaviruses (HCoV) (10.7%), influenza A and B (8.9%), parainfluenza virus (PIV 1–3) (7.9%), and human metapneumovirus (HMPV) (5.0%). PIC (RV/EV) and RSV were the most prevalent etiological agents of SARI in both cities. The total and age-matched prevalence of RSV, HCoV, and hMPV among SARI children under 5 years old were significantly higher in Beijing than in Shanghai. Different age and seasonal distribution patterns of the viral infections were found between Beijing and Shanghai.

**Conclusions:**

Viral infection was tested and shown to be the most prevalent etiological agent among children with SARI in either the Beijing or the Shanghai area, while showing different patterns of viral and epidemiological profiles. Our findings provide a better understanding of the roles of geographic location and climate in respiratory viral infections in hospitalized children with SARI.

## Background

Acute respiratory infections (ARI) are associated with significant morbidity and mortality worldwide, particularly in children under the age of 5 years [1, 2]. Severe acute respiratory infection (SARI) is the leading cause of hospitalization in children and of febrile episodes in infants younger than 3 months old [3, 4]. The most common cause of SARI in children is viral infections [5–10], including influenza viruses A and B (Flu A/B); respiratory syncytial virus (RSV); adenovirus (ADV); parainfluenza virus (PIV) 1–3; picornavirus (PIC), which mainly includes human rhinovirus (RV) and human enterovirus (EV); human coronaviruses (HCoV), which includes OC43, 229E, NL63, and HKU; human bocavirus (HBoV); and human metapneumovirus (HMPV). Although the viral and epidemiological profiles of pediatric patients with SARI vary among countries [4, 7–15], few studies have comprehensively compared the viral and epidemiological profiles of pediatric patients with SARI in different geographic areas or climate zones within the same country, such as China.

Beijing, the capital of the People’s Republic of China, has a population of more than 21 million. Beijing is located in the North of China on the Pacific Ocean, which stands at the northern tip of the North China Plain. Beijing has a semi-humid continental climate in the warm temperate zone. The spring and autumn are relatively short when compared with the duration of summer and winter. The annual temperature is around 11.8 degree Celsius, January can be considered as the coldest month in Beijing for average temperature at − 4.6 degree Celsius, while July will be the hottest month in Beijing. Shanghai is the largest city in China, with a population of more than 25 million. The city is located in the southeast region of the country and has a subtropical monsoon climate. Shanghai lies on China’s east coast roughly equidistant from Beijing. Shanghai’s climate is classified as humid subtropical and experiences four distinct seasons. Summer temperatures at noontime often hit 35–36 °C (95–97 °F) with very high humidity. Winters are chilly and damp, with northwesterly winds from Siberia can cause nighttime temperatures to drop below freezing. In between, spring can feature lengthy periods of cloudy, often rainy, weather, while Autumn is generally mild to dry and sunny. The city averages 4.8 °C (40.6 °F) in January and 28.6 °C (83.5 °F) in July, for an annual mean of 17.1 °C (62.8 °F).

We previously reported the viral etiology of 370 children hospitalized with SARI in Beijing between May 2008 and March 2010 based on an xTAG® RVP FAST assay [15]. The present study is the first to compare the epidemiology and viral pathogens associated with recent SARI in hospitalized children in Beijing and Shanghai between March 2008 and March 2014 by validated polymerase chain reaction (PCR) or real-time reverse transcription-PCR assays and to confirm the findings by sequencing. The cities of Beijing and Shanghai represent the northern and southern regions of China and the temperate monsoon and subtropical monsoon climate zones, respectively.

## Methods

### Ethics, consent, and permissions

The study protocol was approved by the hospitals’ Ethics Committees and the Chinese Center for Disease Control and Prevention. Participants received a document entitled “Written Informed Consent” describing the study’s purpose and their right to keep information confidential. Written consent was obtained from all of the participants or their guardians.

### Patients and specimens

SARI surveillance was conducted in Beijing Children’s Hospital in Beijing and the Children’s Hospital of Fudan University in Shanghai, China between May 2008 and March 2014. All of the patients included in the study were younger than 14 years of age and were diagnosed with SARI according to the World Health Organization (WHO) case definition of a history of symptoms for ≤72 h [4, 5, 15]. Eligibility and classification of the clinical syndromes of SARI were determined from each individual’s original record of medical history and examination. The criteria for inclusion of hospitalized patients in this study were: sudden onset of fever > 38 °C and cough or sore throat and difficulty breathing (dyspnea, oxygen saturation < 90%). To reduce or avoid the inclusion of bacterial causes in children with SARI, additional criteria were a normal or low leukocyte count, or indrawing of the lower chest wall.

We collected 700 nasopharyngeal aspirates (NPAs) between May 2008 and March 2014. There were 259 NPAs from inpatients admitted to Beijing Children’s Hospital, the largest pediatric hospital in northern China, and 441 from children admitted to the Children’s Hospital of Fudan University, the largest pediatric hospital in southeast China. It should be noted that several of the SARI patients in our study resided in areas around Beijing and Shanghai. Informed consent was obtained from the parents of all of the participants before samples were collected. NPAs were collected on the day of admission, placed in a viral transport medium, and stored at − 70 °C prior to analysis. Basic demographic and clinical data were obtained from a questionnaire completed on admission.

### Detection of respiratory viruses by reverse transcription polymerase chain reaction

Nucleic acid was extracted from the samples using QIAamp MiniElute Virus Spin kits (Qiagen, Mississauga, Ontario, Canada) following the manufacturer’s protocol. cDNA was synthesized from 10 μL RNA eluted using the Promega Reverse Transcription System with random hexamer primers and avian myeloblastosis virus reverse transcriptase (Promega, Madison, WI, USA) as described previously [15, 16].

All of the specimens were screened for Flu A/B, PIV1–3, RSV, PIC (RV/EV), and ADV using three validated multiple-nested polymerase chain reaction (PCR) assays [16, 17]. The assays were performed with two rounds under the following conditions. The first round consisted of 94 °C for 5 min; then 94 °C for 30 s, 55 °C for 30 s, and 72 °C for 1 min, 35 cycles; and then 72 °C for 5 min. The second round consisted of 94 °C for 5 min; then 94 °C for 30 s, 50 °C for 30 s, and 72 °C for 1 min, 25 cycles; and then 72 °C for 5 min. The HCoV (including HCoV-OC43, HCoV-229E, HCoV-NL63, and HCoV-HKU1) and HMPV were detected by nested one-step RT-PCR, as described previously [15–17]. The nested one-step RT-PCR was performed in two rounds as follows. The first round consisted of 50 °C for 30 min and then 95 °C for 15 min; then 94 °C for 40 s, 52 °C for 40 s, and 72 °C for 40 s for 35 cycles; and then 72 °C for 5 min. The second round consisted of 94 °C for 5 min; then 94 °C for 40 s, 55 °C for 40 s, and 72 °C for 40 s for 25 cycles; and then 72 °C for 5 min. HBoV was detected using a nested PCR method as described previously [6]. The nested PCR programs were performed in two cycles as follows. The first round consisted of 94 °C for 5 min; then 94 °C for 45 s, 55 °C for 45 s, and 72 °C for 1 min for 35 cycles; and then 72 °C for 5 min. The second round consisted of 94 °C for 5 min; 94 °C for 45 s, 55 °C for 45 s, and 72 °C for 1 min for 25 cycles; and then 72 °C for 5 min. All of the detection assays were validated and optimized to ensure reproducibility, specificity, and sensitivity. Furthermore, all of the positive PCR products were confirmed by sequencing.

### Statistical analysis

Statistical differences were determined using the Chi-square test using SAS software (version 9.2). *P*-values < 0.05 were considered to indicate statistical significance.

## Results

### Patient demographic and clinical characteristics

The study included 700 NPAs from pediatric cases diagnosed with SARI: 259 from the Beijing Pediatric Research Institute of the Affiliated Beijing Children’s Hospital, Capital Medical University (age range, 1 month to 6 years 2 months), and 441 specimens from the Children’s Hospital of Fudan University in Shanghai (age range, 10 days to 14 years). The male-to-female ratio did not differ between hospital samples. We noticed that the difference in the gender ratio (M/F) which is in the range of 1.5–1.8:1.for two groups,the reason need to be clarified on why there are more infected males than females for respiratory infections. The patients were divided into five groups according to age: 0 to 6 months (M), 7 M to 1 year (Y), 1 to 2 Y, 3 to 5 Y, and more than 5 Y (> 5 Y). The mean and median ages in months of the Beijing group were younger than those in the Shanghai group, with more cases in Beijing in the 0–1 Y group and fewer cases in Beijing in the >5Y group. The pediatric SARI cases from Beijing and Shanghai shown different clinical manifestations. The main symptoms found in our study group were cough and fever(≥38 °C), followed by wheezing and diarrhea, runny nose and cyanosis was also recorded. The pediatric SARI cases from Beijing presented a higher frequency of bronchopneumonia and lower Pneumonia when compared to those from Shanghai. A summary of the demographic and clinical characteristics of the participants is presented in Table 1.

**Table 1:** Demographic characteristics of the pediatric SARI cases

Variable	Beijing	Shanghai	*P*-value
	*n* = 259 (%)	*n* = 441 (%)	
Gender ratio (M/F)	1.5:1	1.8:1	0.30730
Age in months (mean/median)	13.1/7	25.9/12	< 0.0001
Age group			
0–6 M	116 (44.8)	139 (31.5)	0.00043
7 M–12 M	51 (19.7)	68 (9.7)	0.14634
1 Y–2 Y	48 (18.5)	74 (16.8)	0.55525
3 Y–5 Y	41 (15.8)	103 (23.4)	0.01740
Clinical manifestations			
Fever(≥38 °C)	155 (59.8)	339 (76.9)	< 0.0001
Cough	250 (96.5)	422 (95.7)	0.66711
Wheezing	54 (20.8)	181 (69.9)	< 0.0001
Runny nose	49 (18.9)	nr	
Diarrhea	17 (6.6)	85 (19.3)	< 0.0001
Cyanosis	nr	49 (11.1)	
Clinical diagnosis			
Bronchopneumonia	117 (45.2)	42 (9.5)	< 0.0001
Pneumonia	123 (47.5)	370 (83.9)	< 0.0001

Note: *nr* No record

### Viral infection profiles

The viral infection profiles are shown in Table 2. Of the 700 NPAs, 547 (78.1%) tested positive for one or more viral pathogens. Single infections were found in 43.9% (307/700) of the cases, and co-infections were found in 34.3% (240/700). The percentage of cases with co-infections in the Beijing sample (47.5%) was significantly higher than that in the Shanghai group (26.5%; *P* = 0.0012). The most frequently detected respiratory virus was PIC (RV/EV), with a prevalence rate of 34.4% (241/700), followed by RSV and HBoV (28.3 and 19.1%, respectively). HCoV was detected in 75 patients (10.7%), ADV was detected in 96 (13.7%), PIV1–3 was detected in 55 (7.9%), Flu A/B was detected in 62 (8.9%), and HMPV was detected in 35 patients (5.0%). The comparison of the respiratory virus detection rates in Beijing and Shanghai revealed that the prevalence of RSV, HCoV, and HMPV were higher in Beijing than in Shanghai.

**Table 2:** Viral infection profiles in paediatric patients with SARI in Beijing and Shanghai

Viruses	Total	Beijing	Shanghai	*P*-value
	*n* = 700	*n* = 259	*n* = 441	
RSV [n (%)]	198 (28.3)	137 (52.9)	61 (13.8)	< 0.0001
RV/EV [n (%)]	238 (34.0)	90 (34.7)	148 (33.6)	0.7485
HBoV [n (%)]	134 (19.1)	56 (21.6)	78 (17.7)	0.2014
HCoVs [n (%)]	75 (10.7)	49 (18.9)	26 (5.9)	< 0.01
ADV [n (%)]	96 (13.7)	31 (11.9)	65 (14.7)	0.3037
PIV 1–3 [n (%)]	55 (7.9)	19 (7.3)	36 (8.2)	0.6945
Flu A/B [n (%)]	62 (8.9)	23 (8.9)	39 (8.9)	0.9868
HMPV [n (%)]	35 (5.0)	22 (8.5)	13 (2.9)	0.0012
Co-infection [n (%)]	240 (34.3)	123 (47.5)	117 (26.5)	0.0012

### Age distribution of viral infections

**Fig. 1:**
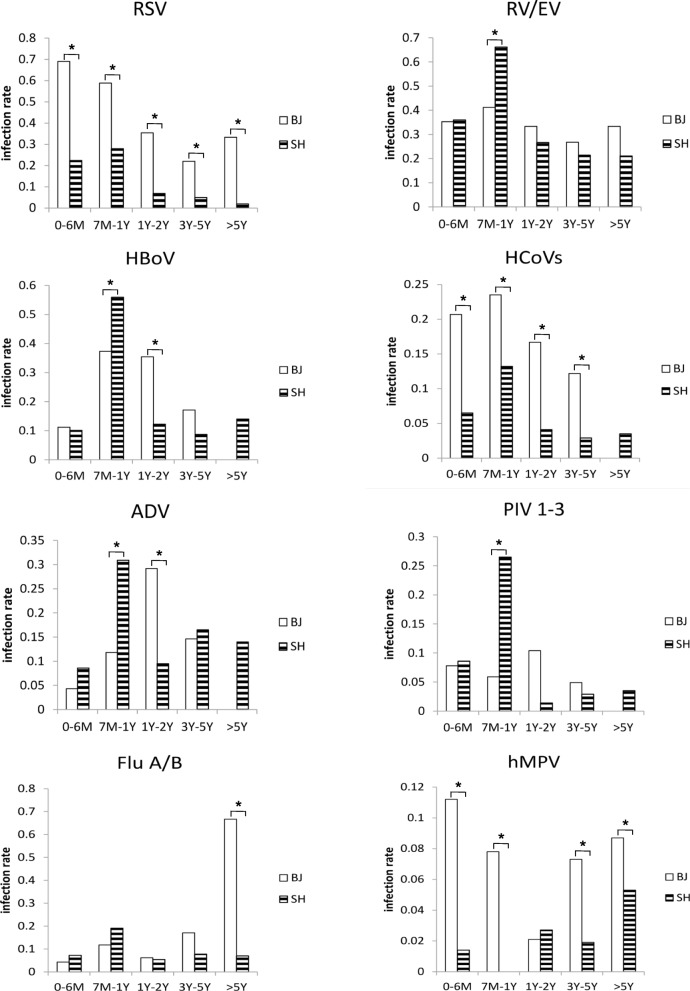
Detection frequencies of respiratory viruses in Beijing and Shanghai according to matched age groups. We considered *P* <  0.05 to be significant (marked as an asterisk) for all analyses between two cities

The distributions of viral infections in Beijing and Shanghai are shown in Fig. 1 according to age groups. All of the viruses were detected in all age groups; however, the prevalence in Beijing and Shanghai differed according to age group. The respiratory virus infection rate in children younger than 1 year old was higher in Beijing than in Shanghai (*P* <  0.05). All of the age-matched distribution patterns for RSV in Beijing were higher than those in Shanghai, and most age-matched distribution patterns in Beijing were higher than those in Shanghai for both HCoV (except for the > 5 Y group) and HMPV (except for the 1–2 Y group). However, the prevalence of PIV 1–3 in Shanghai for the 7 M–1 Y group was significantly higher than that in Beijing. In addition, the peak of individual viral infection differed between Beijing and Shanghai: Flu A/B peaked in the > 5 Y group in Beijing. The ADV infections peaked in the 1–2 Y group in Beijing and in the 7 M–1Y group in Shanghai. The HMPV infections peaked in the 0–6 M group in Beijing and in the > 5 Y group in Shanghai. Moreover, RV/EV, HBoV, and PIV1–3 peaked in the 7 M–1 Y group in Shanghai.

### Seasonality

**Fig. 2:**
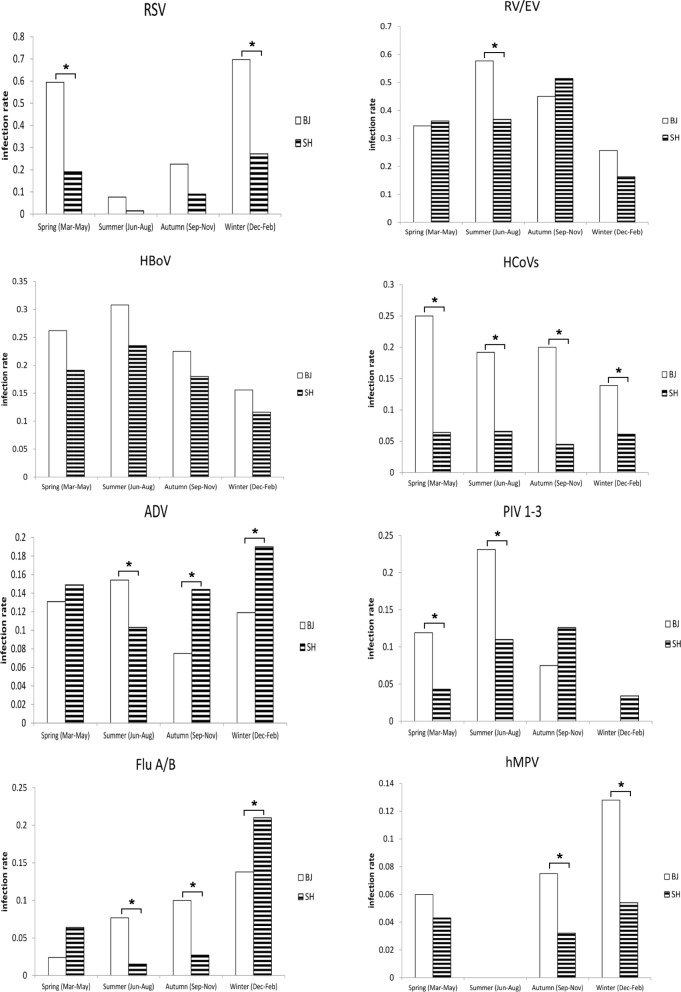
Seasonal distributions of respiratory viruses in Beijing and Shanghai. We considered *P* <  0.05 to be significant (marked as an asterisk) for all analyses between two cities

Comparisons of the seasonal patterns of respiratory viral pathogens in Beijing and Shanghai are shown in Fig. 2. PIC (RV/EV), HBoV, HCoV, and ADV caused infections throughout the year; thus, a seasonal distribution was not apparent in Beijing or Shanghai. RSV peaked in the spring and winter, and Flu A/B and HMPV peaked in the winter in both Beijing and Shanghai. PIV1–3 occurred mainly in the spring and summer in Beijing, whereas this virus was most prevalent in summer and autumn in Shanghai. Interestingly, no SARI cases linked to HMPV infection were detected in the summer in Beijing or Shanghai.

## Discussion

ARI is common in children and can cause mild-to-severe disturbances, including upper and lower respiratory infections, such as pneumonia, bronchiolitis, asthma, and acute respiratory distress syndrome [1, 2]. Our study is the first to compare respiratory viral infections and their epidemiology in hospitalized children with SARI in Beijing and Shanghai. Of the 700 patients included in the study, 78.1% tested positive for at least one virus; this rate was higher than that reported by previous studies (range, 34.6–70.3%) [18–25]. This finding may be mainly explained by some differences in the SARI case inclusion criteria, since additional criteria in our study were a normal or low leukocyte count or indrawing of the lower chest wall. In addition, several factors may account for this disparity, including true differences in the overall burden or differences in study populations or methodologies [18, 23–25]. Moreover, infection rates vary with geographical location and season [22, 26, 27]. Finally, the specific viruses included in our screening decisions may account for the higher positive rates in our study. The positive infection rate in Beijing was slightly higher than that in Shanghai (92.7 vs. 70.1%, respectively). Given the small number of cases and the limited testing period, further studies are needed to determine whether infection rates significantly differ in the two cities.

Previous studies have reported that RSV was the primary cause of SARI in hospitalized children [28–30]. In contrast, PIC (34.0%) was the most common pathogen in our sample, although the findings in Beijing and Shanghai differed. RSV (52.9%) was the leading cause of SARI in Beijing, followed by PIC. However, PIC (33.6%) was the most common cause of SARI in Shanghai, followed by HBoV. Either the total or age group-matched positive rates of RSV, HCoV, and HMPV significantly differed in Beijing and Shanghai (*P* <  0.05). In addition, a comparison of our data with those from other studies collected during the same period in the same locations revealed that our infection rates differed from those of the other studies [15, 19, 25]. Differences in detection methods and primers, sample collection, and subject populations may account for this disparity.

The age distribution patterns of the respiratory viral infections significantly differed in Beijing and Shanghai. In children less than 1 year old, the positive detection rates of several viruses (RSV, HCoV, and HMPV) in Beijing were significantly higher than in Shanghai, whereas the infection rates of other viruses (RV/EV, HBoV, ADV, PIV1–3, and Flu A/B) in Shanghai were significantly higher in Beijing. Given the high co-infection rate in our study, we did not investigate associations between clinical characteristics and individual viral infections. A previous study found that RSV infections were more strongly associated with comorbidities and bronchiolitis than were non-RSV infections [29]. However, it is not clear whether symptoms can be used to identify specific viral infections, and we concluded that no individual symptoms were specific to any viral infection.

Previous investigation also reported that the profiles of respiratory viruses in different area and seasons may be influenced by weather conditions (temperature, humidity) and indoor crowding during the cold season [22, 31]. In this study, the seasonal distribution of viral infections differed in Beijing and Shanghai. PIC infections were more frequent during the summer months in Beijing and more common during autumn in Shanghai, which is consistent with previous findings [15, 19, 25]. Moreover, our findings that RSV and HMPV were prevalent in the spring and winter and that Flu A/B peaked in winter are consistent with those of previous studies [15, 25, 32, 33]. The HCoV epidemic season occurred in the spring in Beijing, whereas the virus did not show a significant seasonal pattern in Shanghai. It may be that HCoV infections occur as biennial outbreaks in Shanghai [16, 34–37]. Previous studies have shown that, although HBoV infections occur throughout the year, they are most evident during the winter and spring months [6, 38, 39]. In contrast, we found that HBoV infections were most common during the summer months in Beijing and Shanghai. We did not have sufficient data to confirm whether climate and geographic location were associated with the virus infection patterns.

Notably, we observed that non-influenza respiratory viruses were common in hospitalized children with SARI. The detection rate of at least one virus was 78.1%. PIC (RV/EV), RSV, HBoV, ADV, and HCoV were the most common pathogens detected, whereas Flu A/B, PIV 1–3, and HMPV were relatively rare in our sample. The total and age-matched prevalence of RSV, HCoV, and HMPV among SARI children under 5 years old were significantly higher in Beijing than in Shanghai. Moreover, the seasonal distributions of the pathogens differed between the regions. We noticed that the mean and median ages in months of the Beijing group were younger than those in the Shanghai group, so age adjustments of the results should be used to clarify future findings based on future larger surveillance studies.

To our knowledge, our study is the first to compare the profiles of multiple (about 15) viruses and their related epidemiological profiles in pediatric patients with SARI in China. Viral infection was tested and shown to be the most prevalent etiological agent among children with SARI in either the Beijing or the Shanghai area, while showing different patterns of viral and epidemiological profiles. The observed pattern of seasonal variation of respiratory viruses are complex between hospitalized children with SARI in Beijing and Shanghai, since the weather and temperature variations between the two cities. Our findings provide a better understanding of the roles of geographic location and climate in respiratory viral infections in hospitalized children with SARI. In addition, our findings provide baseline data for investigations of the burden of respiratory viral infections in Beijing and Shanghai. However, additional studies with larger patient populations are needed to clarify the roles of viral and bacterial pathogens in SARI cases and to evaluate the overall burden of respiratory pathogens in asymptomatic children [40].

## Conclusion

Viral infection was tested and shown to be the most prevalent etiological agent among children with SARI in either the Beijing or the Shanghai area, while showing different patterns of viral and epidemiological profiles. Our findings provide a better understanding of the roles of geographic location and climate in respiratory viral infections in hospitalized children with SARI.

## Data Availability

The datasets used/or analyzed during the current study available from the corresponding author on reasonable request.

## Abbreviations

ADV: Adenovirus
ARI: Acute respiratory infections
EV: Enterovirus
Flu: Influenza
HBoV: Human bocavirus
HCoV: Human coronaviruses
HMPV: Human metapneumovirus
NPA: Nasopharyngeal aspirates
PCR: Polymerase chain reaction
PIC: Picornaviruses
PIV: Parainfluenza virus
RSV: Respiratory syncytial virus
RV: Rhinovirus
SARI: Severe acute respiratory infection
WHO: World Health Organization
